# Latent Epstein–Barr Virus Infection Correlates with Glycosphingolipid Enrichment and Morphological Remodeling of Extracellular Particles

**DOI:** 10.3390/biology15161421

**Published:** 2026-08-18

**Authors:** Alen Zollo, Lucia Centofanti, Valentina Citro, Andrea Corona, Alessandra Mingione, Corinne Monzani, Domenica Giannandrea, Elisa Adele Colombo, Graziano Serrao, Cristina Gervasini, Valentina Massa, Antonino Neri, Alberto Priori, Monica Rosa Miozzo, Paola Signorelli, Raffaella Chiaramonte, Michele Dei Cas, Filippo Martinelli-Boneschi

**Affiliations:** 1 Department of Health Sciences, University of Milan, 20142 Milan, Italy; lucia.centofanti@unimi.it (L.C.); valentina.citro@unimi.it (V.C.); andrea.corona@unimi.it (A.C.); alessandra.mingione@unimi.it (A.M.); corinne.monzani@unimi.it (C.M.); domenica.giannandrea@unimi.it (D.G.); elisaadele.colombo@unimi.it (E.A.C.); graziano.serrao@unimi.it (G.S.); cristina.gervasini@unimi.it (C.G.); valentina.massa@unimi.it (V.M.); alberto.priori@unimi.it (A.P.); monica.miozzo@unimi.it (M.R.M.); raffaella.chiaramonte@unimi.it (R.C.); michele.deicas@unimi.it (M.D.C.); 2 CRC “Aldo Ravelli” for Experimental Brain Therapeutics, Department of Health Science, University of Milan, 20142 Milan, Italy; paola.signorelli@unimi.it; 3 Department of Medicine and Surgery, University of Insubria, 21100 Varese, Italy; 4 Scientific Directorate, Azienda USL-IRCCS of Reggio Emilia, 42122 Reggio Emilia, Italy; antonino.neri@ausl.re.it; 5 Clinical Neurology Unit, Azienda Socio-Sanitaria Territoriale Santi Paolo e Carlo and Department of Health Sciences, University of Milan, 20142 Milan, Italy; 6 Biochemistry Laboratory, IRCCS Policlinico San Donato, 20097 Milan, Italy

**Keywords:** Epstein Barr virus (EBV), *EBER1*, Gb3, ceramide, sphingolipid, extracellular particles

## Abstract

Epstein–Barr virus (EBV) is causally associated with various malignancies and autoimmune diseases. It establishes a lifelong latent infection in host B-lymphocytes, and can strategically manipulate host cell metabolism and cell-to-cell communication via extracellular particles (EPs). While EBV-induced changes in fatty acid and mevalonate pathways are documented, the role of the sphingolipid network in this process remains poorly understood. In this study, we characterized both the EP and the cellular sphingolipid signatures of three EBV-infected (EBV+) lymphoblastoid cell lines compared to three EBV-negative (EBV−) counterparts. Lipidomic analysis revealed a profound metabolic redirection in EBV+ cells, characterized by reduced ceramide and sphingomyelin levels, and a significant upregulation of glycosphingolipids (GSLs), particularly hexosylceramide and globotrialosylceramide. Furthermore, EBV+ cells released larger, more heterogeneous EPs that are significantly enriched in GSLs and in the viral non-coding RNA *EBER1*. Our findings suggest that EBV-induced reshaping of sphingolipid metabolism correlates with altered physical properties of the secretome, potentially contributing to viral pathogenesis and microenvironmental manipulation.

## Simple Summary

Epstein–Barr virus infects the vast majority of the world’s population and, after the initial infection, persists silently for life inside B-lymphocytes. Although usually dormant, this virus is causally linked to several cancers and autoimmune diseases, including multiple sclerosis. In this study, we investigated how the virus, during its dormant phase, reshapes the sphingolipid composition of infected cells and of the membrane-enclosed particles these cells release to communicate with neighboring cells. We found that infected cells show a sphingolipid metabolism skewed toward the overproduction of complex sphingolipid molecules at the expense of simpler ones. The small particles released by infected cells were larger, more heterogeneous, and enriched in these complex sphingolipids. Importantly, these particles also carried viral molecules, including genetic material known to trigger inflammatory and potentially harmful responses in recipient cells, suggesting they may act as vehicles for spreading viral signals throughout the body. These findings reveal a layer of viral manipulation and may help identify new targets for therapies against EBV-associated cancers and immune diseases.

## 1. Introduction

Epstein–Barr virus (EBV) is a ubiquitous human gamma-herpesvirus that infects approximately 90–95% of the global population [1], establishing a lifelong latent infection within host B-lymphocytes [2]. While typically asymptomatic, EBV persistence is causally linked to various malignancies, including B-cell lymphomas and nasopharyngeal carcinoma (NPC) [3], accounting for over 200,000 new cancer cases annually [4]. Furthermore, EBV is recognized as a significant risk factor for several autoimmune diseases [5]; most notably, EBV infection increases the risk of developing multiple sclerosis by 32-fold [6].

To sustain its life cycle and oncogenic potential, EBV strategically manipulates host B-cell metabolism [7], rapidly reprogramming over 11,000 genes to induce pathways necessary for growth and proliferation [8]. A critical focal point of this metabolic rewiring is the sphingolipid network. Recent evidence indicates that an increase in intracellular ceramide (Cer) can trigger EBV lytic replication, an effect that can be reversed by promoting Cer disposal [9]. Beyond Cer, globotriaosylceramide (Gb3) has emerged as a molecule of interest; as a key signaling mediator in germinal center B cells [10], Gb3 promotes B-cell receptor signaling and facilitates T-cell activation [11]. The upregulation of lipogenesis during EBV-driven transformation [12,13] provides the biosynthetic framework to modulate specific species like Gb3, potentially fine-tuning immune engagement and viral persistence.

During latency, EBV expresses a restricted set of viral proteins and short non-coding RNAs (sncRNAs), such as *EBER1*, *EBNA2*, and *LMP1*, which are essential for the conversion of resting B cells into proliferating lymphoblastoid cell lines (LCLs) [14]. This transformation is accompanied by an altered secretome, through which the virus modulates the microenvironment [15]. Cell-to-cell communication is mediated by extracellular particles (EPs), a heterogeneous population comprising extracellular vesicles (EVs) and non-vesicular macromolecular aggregates [16,17].

Consistent with the Minimal Information for Studies of Extracellular Vesicles (MISEV) guidelines, which acknowledge the difficulty in achieving absolute purity from non-vesicular components, we will refer to the isolated material as EPs throughout this manuscript [18].

In EBV-infected cells, host cellular pathways are actively hijacked to release EPs enriched with viral factors like *EBER1* transcript [15,19]. The sphingolipid composition of these particles is crucial for biogenesis, stability and the role in intercellular communication [20]; for instance, Cer is essential for the biogenesis and sorting of the oncoprotein *LMP1* into vesicular cargo [21,22], while sphingosine-1-phosphate (S1P) exerts pro-survival and pro-inflammatory functions [23,24].

Despite these insights, how EBV-induced metabolic reprogramming specifically reshapes both the host cellular sphingolipid profile and its extracellular secretome remains to be fully elucidated. Understanding the balance between different sphingolipid species is essential to clarify how the virus modulates the physical properties and signaling potential of the released EPs.

In the present study, we first aimed to characterize the intracellular sphingolipid signatures and metabolic reprogramming occurring within EBV-infected (EBV+) cell lines compared to EBV-negative (EBV−) counterparts. Subsequently, we investigated how these cellular alterations impact the secretome by integrating lipidomic characterization, metabolic ratio analysis, and viral transcript profiling of the released EPs. By combining these molecular data with particle size analysis, we define how EBV-driven sphingolipid changes reshape the physical and functional identity of the EPs, ultimately contributing to viral pathogenesis and microenvironmental manipulation.

## 2. Materials and Methods

### 2.1. Cell Lines

Three cell lines derived from multiple myeloma were selected for the present study: CMA-03 [25], CMA-03/06 [26] and OPM2; all three cell lines tested negative for EBV (EBV−). Three LCLs were used, namely ARH77 (multiple myeloma-derived), CN1208550M and CN50271, as EBV-positive cells (EBV+). OPM2 and ARH77 were purchased from ATCC. CMA-03 and CMA-03/06 were kindly provided by prof. Neri [25]. CN1208550M and CN50271 were obtained from the Telethon Network of Genetic Biobanks (Gaslini Genetic Bank, Genova, Italy) [27], were mycoplasma-tested, and their use was approved by Ethical Committee of Università degli Studi di Milano (Comitato Etico No. 99/20, 17 November 2020). We acknowledge that this multi-line approach compares biologically distinct B-cell systems rather than an isogenic model. However, evaluating multiple independent cell lines within the same B-cell lineage allows us to identify recurrent metabolic trends that transcend specific cell-line backgrounds, providing valuable preliminary screening insights into EBV-driven alterations.

All cell lines were cultured in a humidified incubator at 37 °C with 5% CO2. CMA-03 cells were maintained in Iscove’s modified Dulbecco’s medium (IMDM) (EuroClone, Milan, Italy) supplemented with 10% fetal calf serum (FCS) (EuroClone) and 20 U/mL recombinant human interleukin-6 (IL-6) (R&D System, Minneapolis, MN). CMA-03/06 cells were cultured under identical conditions except for the absence of IL-6.

OPM2, ARH77, CN1208550M and CN50271 were maintained in Roswell Park Memorial Institute 1640 (RPMI 1640) (EuroClone) supplemented with 10% of fetal bovine serum (FBS, Thermofisher Scientific, Monza, Italy) and 1% penicillin/streptomycin (Thermofisher Scientific).

Cell cultures were maintained at a density ranging between 0.5 and 1.0 × 10^6^ cells/mL to ensure exponential growth. Subculturing was performed every 2–3 days by partial replacement of the medium or by dilution, depending on the specific growth rate of each cell line. Cell viability was routinely monitored using the Trypan Blue assay (Sigma Aldrich, Milan, Italy) and only cultures with viability higher than 95% were used for subsequent experimental procedures.

### 2.2. Extracellular Particle Isolation and Quantification

Conditioned medium from 24 h cell culture underwent a triple centrifugation step at ascending speeds (1000, 2000 and 3000× *g*) for 15 min at 4 °C to eliminate most cell debris and aggregates. Subsequently, supernatant was ultra-centrifuged at 110,000× *g* for 75 min at 4 °C using an Optima MAX-XP Ultracentrifuge (Beckman Coulter, Milan, Italy). The resulting supernatant was carefully removed and the EPs stored at −80 °C for further validation and analysis.

Quantification of EPs isolated from both plasma and supernatant was performed by using nanoparticle-tracking analysis (NTA), namely NanoSight Pro (Malvern Panalytical Ltd., Malvern, UK). For each cell line, EP isolation was performed in at least three independent biological replicates. NTA measurements revealed a consistent average yield across all cell lines, maintaining a concentration of approximately 10^11^ particles/mL.

Since the used isolation method may co-precipitate non-vesicular components (e.g., soluble proteins, lipoprotein particles and ribonucleoprotein complexes), along with extracellular vesicles, we adopt the term EPs rather than EVs to reflect this methodological limitation, in line with MISEV guidelines [18]. In spite of the possible co-precipitates, the consistent presence of intact EVs surrounded by a lipid bilayer was assessed by cryo-electron microscopy at the Unitech NOLIMITS facility (University of Milano) on the OPM2 cell line as proof of concept (see Supplementary Materials, Supplementary Figure S1). To validate the vesicular identity of the isolated fraction, Western blot analysis for canonical extracellular vesicles markers (TSG101, CD63, CD81 and CD9) was performed on representative EBV+ and EBV− cell line-derived EPs, as described in the Supplementary Materials. The characterization data are provided in Supplementary Figure S2.

### 2.3. RNA Extraction, TaqMan Probes and Real-Time Quantitative PCR

Cells and EPs were lysed in 700 uL of Qiazol (Qiagen, Hilden, Germany) and frozen at −80 °C upon extraction. Total RNA extraction was performed with Rneasy MinElute cleanup kit (Qiagen) as per manufacturer instructions, including on-column DNase I digestion to remove genomic DNA. RNA quantity and purity were assessed via NanoDrop (Thermo Scientific, Milan, Italy).

TaqMan probes for *EBER1*, *EBNA1*, *EBNA2 type1*, *EBNA2 type2*, *LMP1*, *LMP2a* were obtained from Thermofisher Scientific based on previously published works [28,29]. Selected housekeeping genes were *OST4* and *TOMM7* [30]. To retrotranscribe mRNA, SuperScript IV VILO Mastermix (Invitrogen, Milan, Italy) was used as per the manufacturer’s instruction, with 500 ng RNA input. *ZTA* primer was obtained from Sigma Aldrich based on previously published works [31,32] (see Supplementary Materials).

cDNA was quantified with Nanodrop (Thermo Scientific), and 30 ng per 20 uL reaction was amplified with TaqMan Fast Advanced Master Mix (applied biosystem), 250 nM probes, using the following program: 50 °C/2 min, 95 °C/20 s, and then 40 cycles of 95 °C/1 s + 60 °C/20 s.

Relative quantification of gene expression levels (fold change) based on cycle threshold (CT) values was used to examine the data from RT-qPCR. Specifically, target gene expression was normalized to housekeeping genes to obtain ∆CT values. Figure 2 represents the relative fold change calculated using the ∆ ∆CT method, where the ∆CT of LCL cells was used as the reference calibrator.

### 2.4. Sphingolipid Extraction and Analysis

EPs were suspended with water (100 μL), with an added a mixture of internal standards (Cer 12:0, sphingomyelin (SM) 12:0, hexosylceramide (HexCer) 12:0, lactosylceramide (LacCer 12:0) at 20 μM in methanol, 10 μL), and extracted with methanol/chloroform (850 μL, 2:1, *v*/*v*). The bulk of esterified fatty acids (e.g., phospholipids, acylglycerols) was hydrolyzed by alkaline methanolysis (75 μL methanolic potassium hydroxide (KOH) 1M) in an oscillator thermo-mixer for 2 h (38 °C, 1000 rotation per minute). Then, samples were neutralized by the addition of glacial acetic acid (4 μL). After centrifugation (30 min at 20,000× *g*, 4 °C), the organic phase was vacuum-evaporated in a SpeedVac concentrator (SPD130DLX-230, Thermo Fisher, Waltham, MA, USA). The residues were dissolved in 100 μL of methanol + 0.5 mg/mL dibutylhydroxytoluene and withdrawn in a glass vial after additional centrifugation. Pure extracts (5 μL) were directly injected in liquid chromatography tandem mass spectrometry (LC-MS/MS). The samples were analyzed by a LC-MS/MS system (AB Sciex 3200 QTRAP, Concord, ON, Canada) equipped with an electrospray ionization source (ESI) source and coupled with LC Dionex 3000 UltiMate (ThermoFisher Scientific, Waltham, MA, USA). Chromatographic separation was carried out on a reverse-phase Acquity UPLC BEH C8 column 1.7 μm particle size, 100 × 2.1 mm (Waters, Franklin, MA, USA) at 30 °C using a linear gradient elution with two solvents: 0.2% formic acid and 2 mM ammonium formate in water (solvent A), and 0.2% formic acid and 1 mM ammonium formate in methanol (solvent B). The elution gradient (%B) was set as follows: 0–3 min (80–90%), 3.0–6.0 min (90%), 6.0–19.0 min (90–99%), 19.0–20.0 min (99–80%), held until 24 min. The flow rate was kept constant at 0.30 mL/min during the analysis. The separated analytes were detected with a triple quadrupole MS operated in multiple reaction monitoring mode via positive ESI [33]. The analytes detected are the sphingolipids with fatty acids from 16:0 to 24:0 belonging to the subclasses of dihydroceramide (DHCer), Cer, SM, HexCer, LacCer, gangliosides GM3, globosides Gb3. The analysis of sphingoide bases was carried out on a Cortecs C18 1.6 μm, 2.1 × 100 mm (Waters, MA, USA), as already reported in [33].

### 2.5. Statistical Analysis

All data in this study were analyzed using GraphPad Prism ver. 8 software (GraphPad Software, San Diego, CA, USA). Sphingolipid absolute abundances were normalized onto total protein concentration of the samples. Sphingolipid abundances and EP data were analyzed using unpaired two-tailed *t*-test or one-way ANOVA, followed by Tukey’s post hoc test, with significance set at 0.05. Given the sample size of *n* = 3 biological replicates per group, formal normality testing was not applied, as such tests have insufficient statistical power at this scale. Prior to each pairwise comparison, the F-test for equality of variances was applied; where significant heteroscedasticity was detected, Welch’s correction was used. Given the exploratory nature of this study, no correction for multiple comparisons was applied; the results should therefore be interpreted as hypothesis-generating.

qPCR results were analyzed with a paired *t*-test, with significance set at 0.05.

## 3. Results

### 3.1. EBV+ Cell Lines Produce Larger EPs

Given the role of EPs in lymphocyte communication and in viral spreading and infection, we assessed the size and quantity of EPs isolated from culture medium and produced from human B-lineage cell lines, comprising multiple myeloma (ARH77, OPM2, CMA-03, CMA-03/06) and lymphoblastoid (CN1208550M and CN50271) lines.

ARH77, CN1208550M and CN50271 are EBV+ and, by contrast, OPM2, CMA-03 and CMA-03/06 were tested EBV-negative.

**Figure 1:**
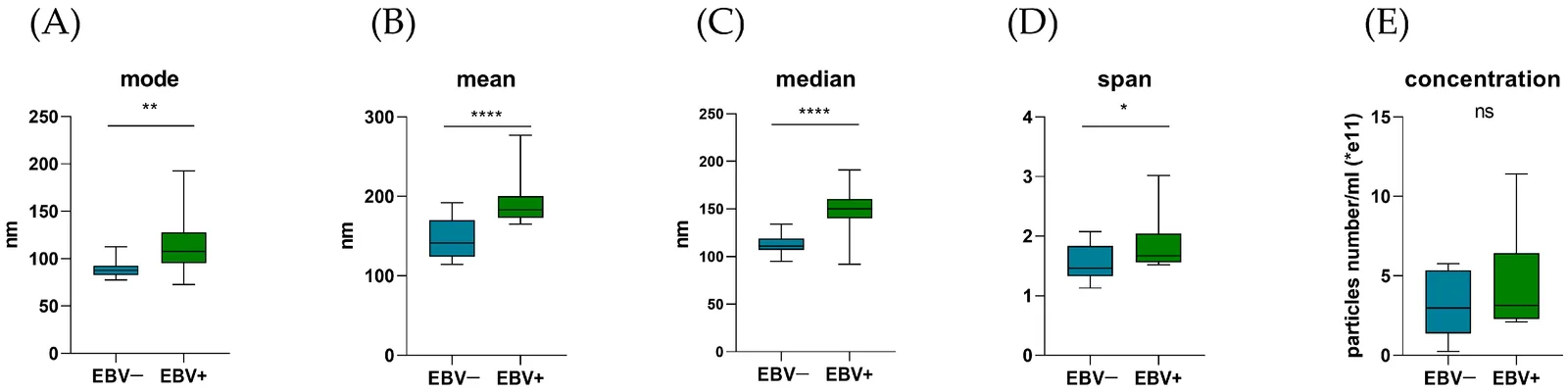
NTA analysis of EPs isolated from EBV+ and EBV− cell lines. EBV+ EPs showed an increase in mode ((**A**), 114.0 ± 29.4 nm vs. 88.17 ± 9.0 nm, *t*-test *p* = 0.0017), mean ((**B**), 190.8 ± 27.4 nm vs. 147.3 ± 24.9 nm, *t*-test *p* = 0.0001), median ((**C**), 149.5 ± 20.8 nm vs. 112.7 ± 10.8 nm, *t*-test *p* < 0.0001) and span ((**D**), 1.854 ± 0.43 vs. 1.557 ± 0.29, *t*-test *p* = 0.0432). The number of particles produced from the different cell lines in identical experimental conditions is comparable ((**E**), 4.4 ± 2.9 vs. 3.1 ± 2.0 particles/mL, *t*-test *p* = 0.1). *, *p* < 0.05; **, *p* < 0.01; ****, *p* < 0.0001; ns, not significant.

The EPs derived from EBV+ cell lines (EBV+EPs), analyzed by nanoparticle-tracking analysis, showed an increase in mode (114.0 ± 29.4 nm vs. 88.17 ± 9.0 nm, *t*-test *p* = 0.0017) (Figure 1A), mean size (190.8 ± 27.4 nm vs. 147.3 ± 24.9 nm, *t*-test *p* = 0.0001) (Figure 1B), median (149.5 ± 20.8 nm vs. 112.7 ± 10.8 nm, *t*-test *p* < 0.0001) (Figure 1C), and span (1.854 ± 0.43 vs. 1.557 ± 0.29, *t*-test *p* = 0.0432) (Figure 1D) if compared to EBV− cell line-derived EPs (EBV−EPs). In NTA analysis, the statistical mode identifies the diameter of the most represented particle population, while the span index quantifies sample heterogeneity. The concomitant increase in both parameters in EBV+EPs indicates not only that the predominant vesicle class is structurally larger, but also that overall secretome becomes significantly more polydisperse in particles derived from EBV+ cell lines. The number of EPs produced by the two groups did not differ significantly, although a modest increase was observed in EBV+ samples compared with the EBV− group (4.4 ± 2.9 vs. 3.1 ± 2.0 particles/mL, *t*-test *p* = 0.1) (Figure 1E).

The presence of intact EVs was demonstrated by cryo-electron microscopy (see Supplementary Figure S1) and the vesicular identity of isolates via Western blotting (see Supplementary Figure S2).

### 3.2. LCLs Express EBV Latency Genes and Release EPs Enriched in EBER1

To evaluate the cargo of EBV+EPs and to identify key packaged transcripts in our model, we evaluated the transcription of latency associated genes by measuring their mRNA in the three selected LCLs and their relative EPs.

The expression of the lytic gene *ZTA* was not detected in any sample (see Supplementary Materials), confirming that LCLs were not switching to the lytic phase and maintained the latency stage. Then, we compared the expression of the main genes involved in the viral latency and analyzed their transcripts isolated from the LCLs and relative EPs. Latency gene expression was quantified via TaqMan probes qRT-PCR.

**Figure 2:**
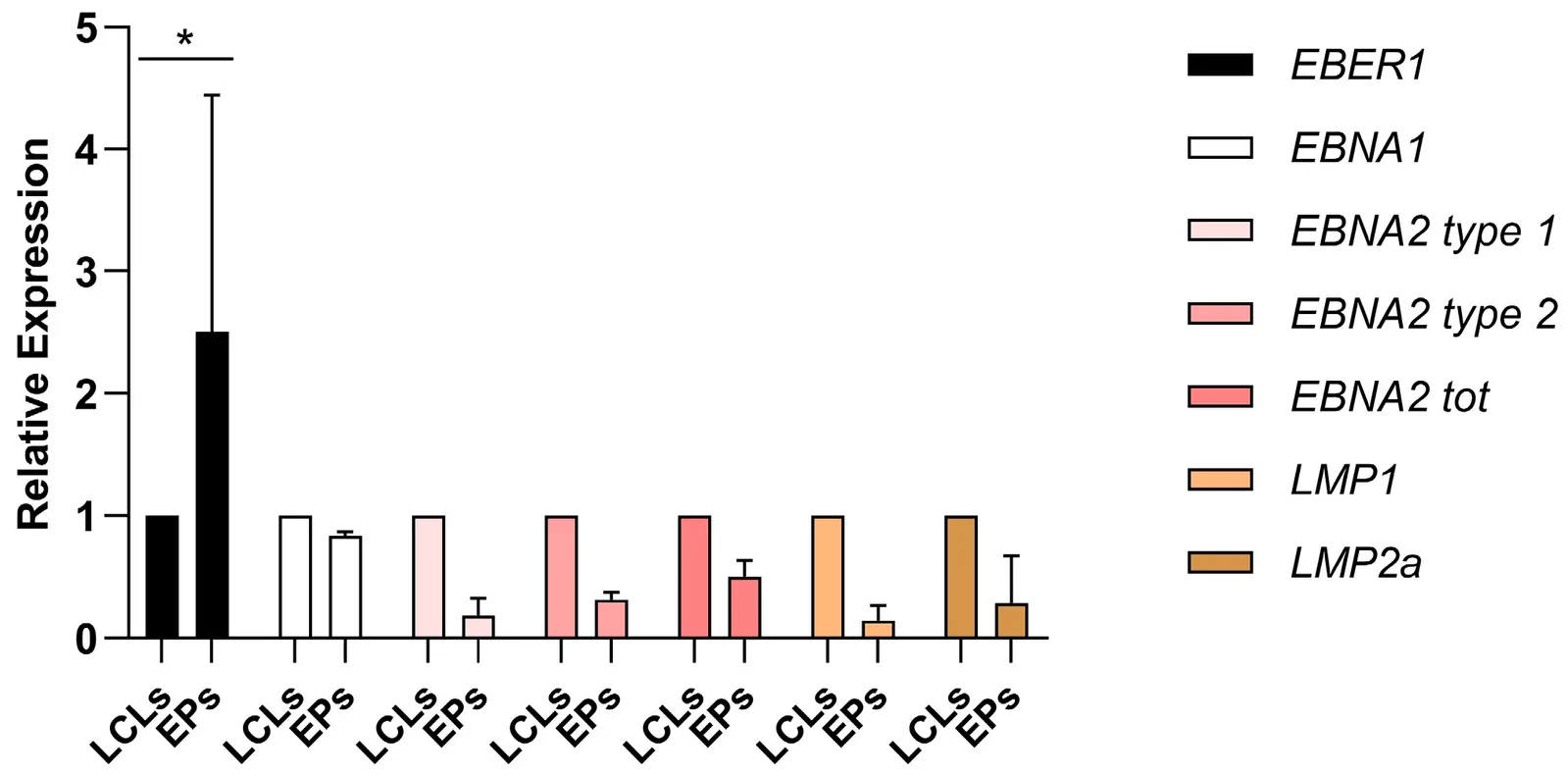
Expression profile of the seven main genes involved in the viral latency in EBV+ cell lines (LCLs) and their derived EPs. The expression of transcripts in cell lines is compared with its amount in the parental EPs. The relative fold change was calculated using the ∆ ∆CT method, where the ∆CT of LCL cells was used as the reference calibrator. *, *p* < 0.05.

Among the analyzed targets, *EBER1*, the only sncRNA among those analyzed, resulted in a higher abundance of EPs compared to cell lines (∆∆CT mean diff. 1.5, *p* < 0.04). On the contrary, we observed that *EBNA1*, *EBNA2*, *LMP1* and *LMP2a* were more abundantly expressed in cell lines than EPs (Figure 2).

Among the viral transcripts analyzed in LCL-derived EPs, *EBER1* consistently displayed the lowest CT values, suggesting a strong enrichment, while transcripts encoding latent membrane proteins (*LMP1*, *LMP2a*) were barely detectable. Due to intrinsic differences in primer amplification efficiencies, these values serve as a descriptive profile of the EP cargo rather than an absolute quantification.

### 3.3. EBV Regulates Sphingolipid Metabolism and Alters EP Composition

To study how viral infection may affect the sphingolipid metabolism in B-lymphocytes, we evaluated the sphingolipid profile of the cell lines and their derived EPs by LC-MS/MS-based targeted analysis.

We analyzed the sphingolipid composition of EBV+ and EBV− cell lines, as well as the derived EPs, selecting three cell lines for each group to ensure robust comparison.

**Figure 3:**
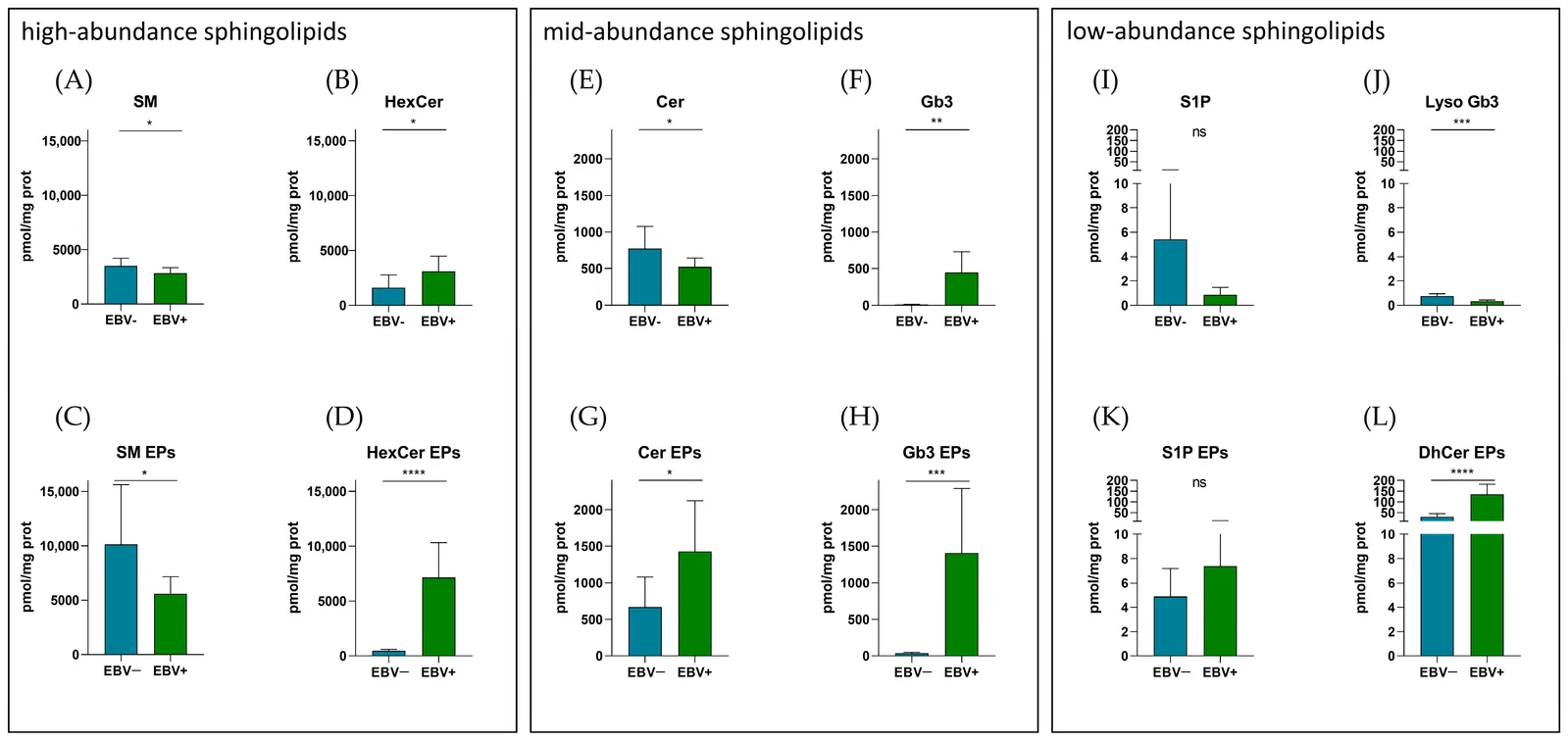
The abundance (pmoles) of sphingolipid species in EPs and their parental cell lines was analyzed via LC-MS/MS. The EBV+ group includes three LCLs, and the EBV− group includes three multiple-myeloma-derived cell lines. Upper graphs represent data from cell lines; bottom graphs represent data from EPs. Data were normalized onto total protein concentration (mg) and a *t*-test was performed. Mean of EBV+ vs. EBV− in pmol/mg prot, (**A**): SM: 3485 vs. 2817, *p* = 0.0474; (**B**): HexCer: 1598 vs. 3082, *p* = 0.0357; (**C**): SM: 10,142 vs. 5589, *p* = 0.0396; (**D**): HexCer: 459.7 vs. 7170, *p* < 0.0001; (**E**): Cer: 774.4 vs. 521.1, *p* = 0.0466; (**F**): Gb3: 10.8 vs. 446.4, *p* = 0.0015; (**G**): Cer: 666.7 vs. 1428, *p* = 0.0185; (**H**): Gb3: 36.4 vs. 1408, *p* = 0.0003; (**I**): S1P: 0.86 vs. 5.42, *p* = 0.066; (**J**): LysoGb3: 0.75 vs. 0.34, *p* = 0.0002; (**K**): S1P: 4.9 vs. 7.4, *p* = 0.229; (**L**): DHCer: 30.8 vs. 135.2, *p* < 0.0001. *, *p* < 0.05; **, *p* < 0.01; ***, *p* < 0.001; ****, *p* < 0.0001; ns, not significant.

We compared the sphingolipid abundances in cell lines and EPs starting from an inter-group comparison between cell lines EBV+ vs. EBV− and between EBV+EPs vs. EBV−EPs (Figure 3), focusing on lipid species that showed statistically significant differences. Comparing EBV+ vs. EBV− cells, we observed a decrease in SM (mean diff. 668.0 ± 309.3 pmol/mg prot, *t*-test, *p* = 0.047), Cer (mean diff. 253.3 ± 116.1 pmol/mg prot, *t*-test *p* = 0.046) and lyso globotriaosylceramide (lyso Gb3) (mean diff. 0.417 ± 0.081 pmol/mg prot, *t*-test *p* = 0.0002), and a concomitant increase in HexCer (1484 ± 638.8 pmol/mg prot, *t*-test *p* = 0.036) and Gb3 (mean diff. 435.5 ± 109.1 pmol/mg prot, *t*-test *p* = 0.0015). S1P is decreased in EBV+ cell lines, without statistical significance.

In EBV+EPs vs. EBV−EPs, there is an increase in DHCer (mean diff. 104.4 ± 17.2 pmol/mg prot, *t*-test *p* < 0.0001), Cer (mean diff. 761.7 ± 285.9 pmol/mg prot, *t*-test *p* = 0.0185), HexCer (mean diff. 6710 ± 1111 pmol/mg prot, *t*-test *p* < 0.0001) and Gb3 (mean diff. 1371 ± 291.1 pmol/mg prot, *t*-test *p* = 0.0003). S1P levels are increased, although not significantly, in contrast to the decrease observed in the corresponding cell lines. On the other hand, SM levels are increased (mean diff. 4553 ± 2020 pmol/mg prot, *t*-test, *p* = 0.0396).

In order to appreciate how the Eps’ lipid component reflects or differs from the cellular lipidome and if such a profile is affected by EBV infection, we conducted an intra-group comparison to assess the extent of selective sorting of sphingolipid species into EPs.

**Figure 4:**
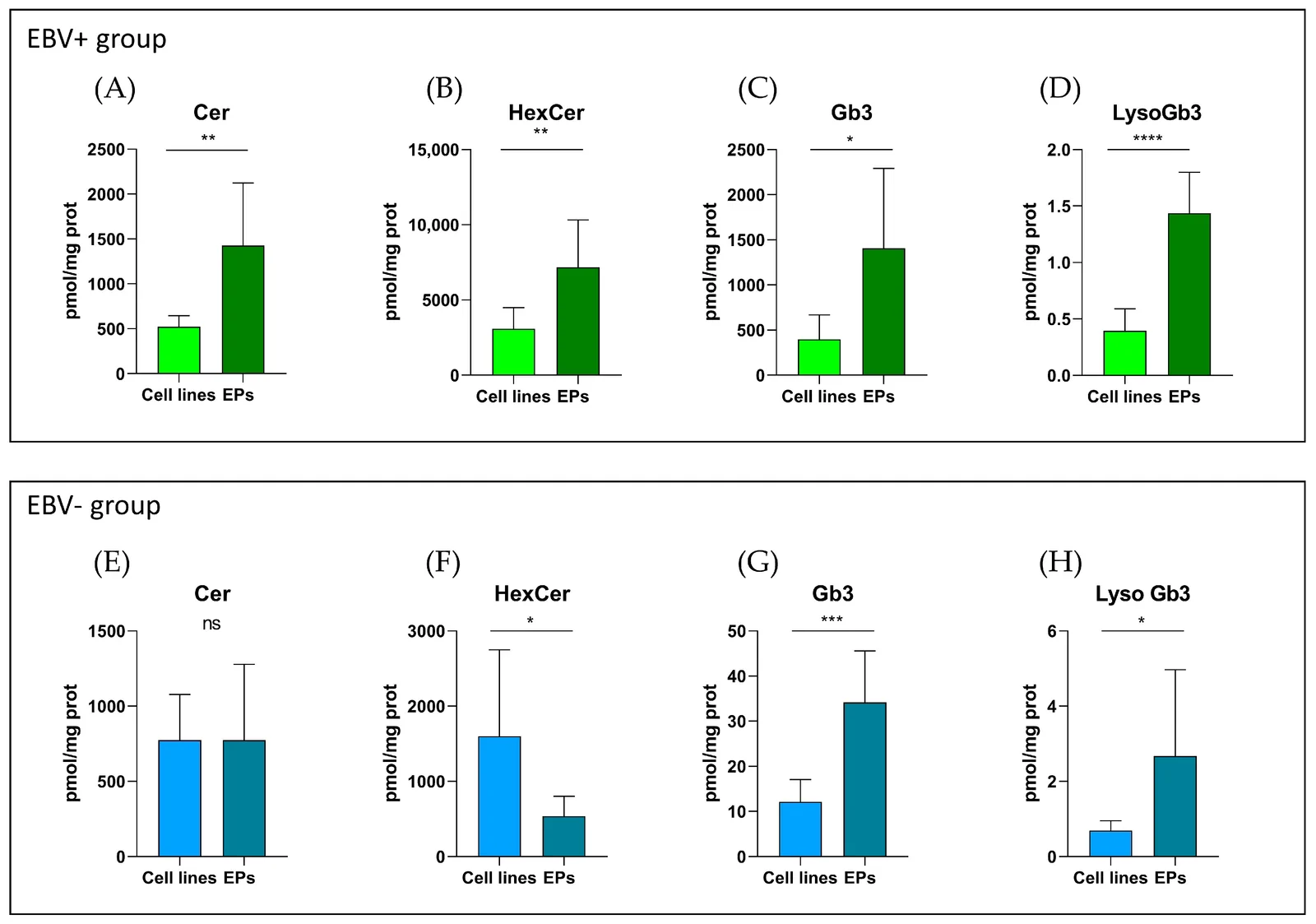
The abundance of sphingolipid in cell lines and EPs was compared separately within the group EBV+ (green bars) and EBV− (blue bars). Data were normalized on mg of proteins and a *t*-test was performed. Mean of cell lines vs. EPs in pmol/mg prot; EBV+ group: (**A**) Cer: 521.1 vs. 1428, *p* = 0.0027; (**B**) HexCer: 3082 vs. 7170, *p* = 0.0087; (**C**) Gb3: 396.9 vs. 1408, *p* = 0.0134; (**D**) LysoGb3: 0.4 vs. 1.4, *p* < 0.0001. EBV− group: (**E**) Cer: 774.4 vs. 666.7, *p* = 0.6; (**F**) HexCer: 1598 vs. 536.3, *p* = 0.0159; (**G**) Gb3: 12.1 vs. 34.1, *p* = 0.0002; (**H**) LysoGb3: 0.7 vs. 2.7, *p* = 0.02. *, *p* < 0.05; **, *p* < 0.01; ***, *p* < 0.001; ****, *p* < 0.0001; ns, not significant.

Comparing the abundance of sphingolipids within the EBV+ group (Figure 4, upper graphs), EPs were found to be enriched in Cer (mean diff. 907.3 ± 249.6, *p* = 0.0027), HexCer (mean diff. 4088 ± 1306, *p* = 0.0087), Gb3 (mean diff. 1011 ± 348.8, *p* = 0.0134) and lyso Gb3 (mean diff. 1.039 ± 0.1453, *p* < 0.0001). When considering its counterpart, EBV−cell lines and EBV−EPs (Figure 4, bottom graphs), Cer levels were found to be unaltered (mean diff. 107.7 ± 181.3, *p* = ns), HexCer was found to be less abundant in EPs (mean diff. 1061 ± 393.7, *p* = 0.0159), and Gb3 (mean diff. 21.99 ± 4.4, *p* = 0.0002) and lyso Gb3 (mean diff. 1.98 ± 0.77, *p* = 0.0203) were determined to be more abundant in EPs.

Thus, EPs were found to be enriched in the Gb3 component if compared to the producing cells, regardless of EBV infection, whereas their sphingolipid precursors are specifically secreted in the EPs of infected cells.

**Figure 5:**
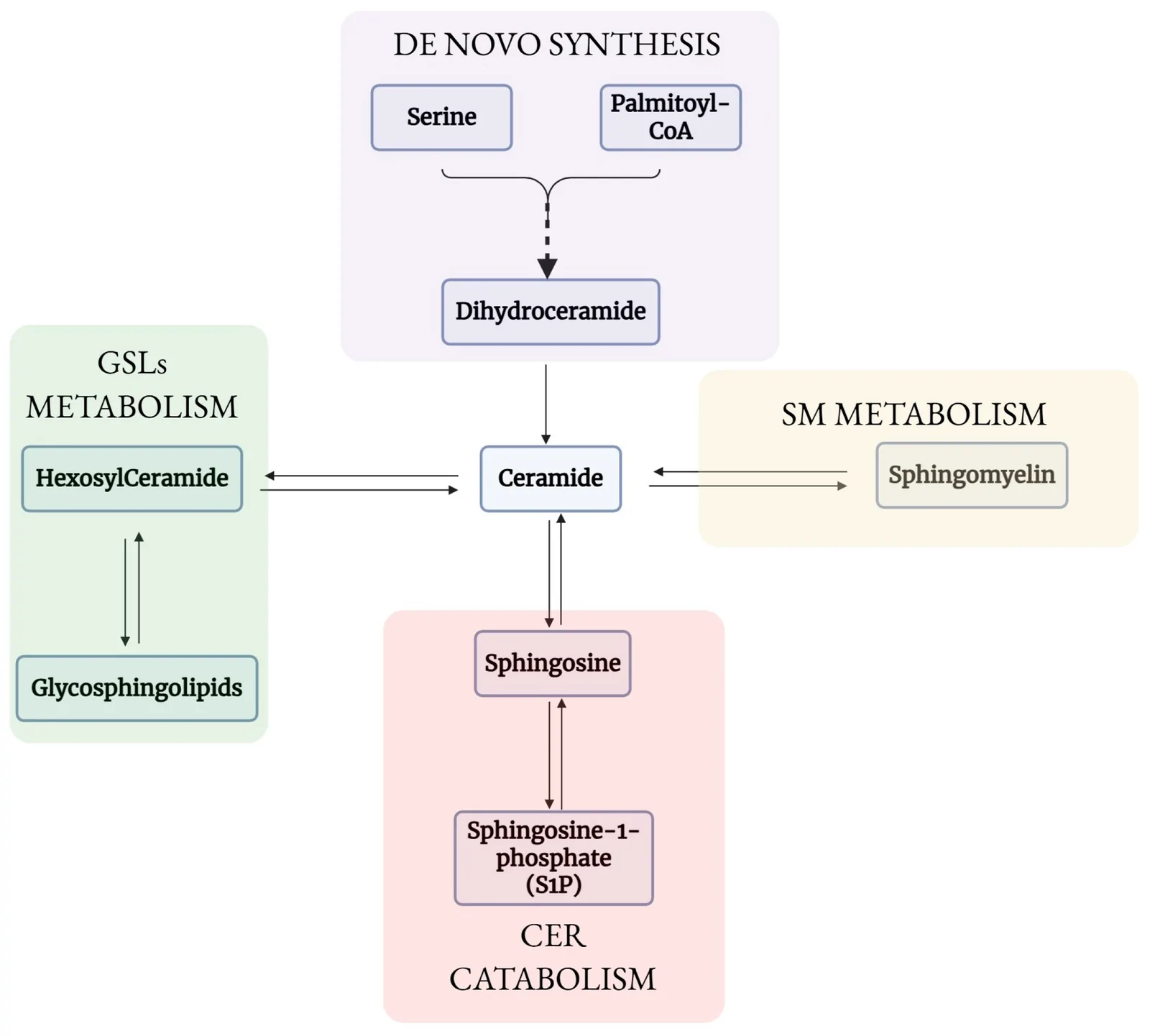
Schematic representation of the sphingolipid metabolic pathway.

The concentration of individual sphingolipid species is subjected to biological fluctuation. Among the identified sphingolipids, there were metabolic intermediates that feed into distinct product pathways (e.g., Cer can be converted into SM by the addition of phosphocholine or glycosylated to form HexCer and other complex GSLs, or degraded to sphingosine (Sph), which can eventually be phosphorylated to S1P; see Figure 5).

Therefore, we calculated sphingolipid ratios to estimate the metabolic trends in EBV-infected and not infected cells. The ratio between selected sphingolipids is reported in Table 1 as ∆ log fold changeThe Cer/SM ratio was found to be decreased in EBV+ cell lines when compared to EBV− cell lines (∆ = 0.9, *p* = 0.0721). The Cer/S1P ratio was found to be decreased when comparing EBV+EPs and their parental cell lines (∆ = −3.6, *p* = 0.001). Interestingly, the Cer/HexCer ratio was reduced in EBV+ cell lines compared with EBV− cell lines (∆ = −3.3, *p* = 0.0427), and a decrease was observed when EBV+EPs were compared with their parental cell lines (∆ = −1.6, *p* = 0.0216). The Gb3/HexCer ratio was increased in EBV+ cell lines compared to EBV− cell lines (∆ = 3.6, *p* = 0.0005), and in EBV+EPs compared both to EBV+ cell lines (∆ = 0.4, *p* = 0.0453) and EBV−EPs (∆ = 2.2, *p* = 0.0002).

## 4. Discussion

The present study investigated the impact of latent EBV infection on sphingolipid metabolism and its potential implications for cell-to-cell communication. By analyzing three EBV+ and three EBV− cell lines, we identified specific sphingolipid signatures that differentiate infected from uninfected cells. While the use of cell lines from different pathological backgrounds (LCLs vs. multiple myeloma) represents a non-isogenic system that requires cautious interpretation, this screening approach successfully outlines clear lipidomic distinctions. The EBV+ cells exhibited reduced levels of SM and Cer and increased amounts of HexCer and Gb3. This profile suggests a metabolic shift toward GSLs biosynthesis at the expense of Cer accumulation.

This metabolic profile may be consistent with the viral strategy of maintaining latency. Indeed, it is known that increased Cer concentration can trigger the transition to the lytic cycle, a process that can be reversed by promoting Cer disposal [9], highlighting the critical role of lipid metabolism in determining viral fate [34]. While the impact of EBV on fatty acid and mevalonate metabolism is well documented [35,36], our data showed that a profound reprogramming of the sphingolipid metabolism occurs during chronic latent infection in B-lymphocytes. In this context, GSL upregulation has been associated with prevention of ceramide-induced lytic reactivation and with support of B-cell proliferation and signaling in published models [7].

The analysis of precursor-to-product ratios provided further mechanistic insights in relation to their biological role (see Table 2). In EBV+ cells, the marked reduction in Cer/DHCer (−3.8) and Cer/HexCer (−3.3) ratios, combined with the increase in the Gb3/HexCer ratio (+3.6), is consistent with a metabolic trend toward enhanced de novo synthesis and glycosylation. This metabolic shunting toward complex GSLs, such as Gb3, mirrors adaptations described in EBV-transformed cells, in which viral proteins coordinate lipogenesis to evade apoptosis [37]. The concomitant decrease in lyso Gb3 and S1P further supports the establishment of an anti-apoptotic state by maintaining low intracellular Cer levels [23,24,38,39].

Our NTA data provide physical evidence of this metabolic remodeling: EPs derived from EBV+ cell lines exhibited a significant increase in both size and polydispersity compared to EBV− counterparts, suggesting a distinct alteration in EP composition [40,41]. Rather than a simple numerical increase, latent infection may prompt the release of diversified vesicular populations, likely required to modulate the microenvironment [15,42].

We hypothesize that this morphological shift may be linked to the altered sphingolipid profile, as lipids are critical in shaping membrane curvature [43]. Specifically, the dynamic ratio between Cer and its glycosylated products influences both the morphology and cargo loading of the released EPs [44,45]. Biophysical studies and molecular dynamics simulations have shown that while Cer favors negative curvature and high-bending membranes due to its cone-like shape [45], higher GSL content (such as HexCer and Gb3) stabilizes less curved, thicker, and more ordered bilayers [46,47]. In our model, EBV+ cells showed a significant shift toward HexCer and Gb3 at the expense of Cer. This GSL enrichment suggests a possible physical framework to correlate the larger and more heterogeneous EPs observed. Furthermore, the higher Cer/SM ratio found specifically in EBV+EPs suggests that the budding process may occur at specific membrane microdomains where Cer is concentrated, a mechanism potentially regulated by *LMP1* to optimize its own packaging into vesicles [21,22]. Notably, while *LMP1* mRNA transcripts were barely detectable in LCL-EPs, the *LMP1* protein is consistently reported as highly enriched in EVs from EBV+ B cells, including LCL [22]. This protein–RNA discrepancy reflects post-transcriptional sorting via Cer-dependent trafficking through CD63+ multivesicular bodies, suggesting EBV+EPs may carry functional *LMP1* protein to activate NF-kB/PI3K signaling in recipient cells.

Thus, our results suggest that EBV is associated with altered sphingolipid metabolism that correlates with the release of larger, GSL-enriched particles.

These metabolic alterations are reflected in the cargo of the released EPs. EPs derived from LCLs were enriched in DHCer, Cer, HexCer, and especially Gb3 compared to their EBV− counterparts.

Gb3 is known to be expressed by LCL and multiple myeloma cells [48]; it plays a functional role in germinal centers for B-cell maturation [10]. The selective concentration of Gb3 within this fraction suggests specific packaging; in published models, Gb3 has been shown to induce the activation of ERK and p38 MAPK signaling pathways in recipient cells, promoting cell proliferation without additional growth factors [49], though whether similar effects occur here remains untested.

Beyond the lipid profile, the EP fraction is characterized by the presence of the small non-coding RNA *EBER1*. Although total *EBER* release may be lower than 25% in some models [19], its consistent detection in LCL-derived EPs in our study raises the possibility that these particles may represent a vehicle for its extracellular dissemination. In published in vitro studies, the number of *EBER1* copies delivered to target cells has been reported to correlate with the induction of interferon- and inflammation-related genes [50], and paracrine immunomodulatory effects of *EBER1*-containing vesicles have been proposed. Whether the *EBER1*-carrying EPs characterized here exert similar effects on recipient cells remains to be established by dedicated functional assays, which are beyond the scope of the present study [19,50,51,52]. Contrariwise, the CT values of *LMP1* transcripts were closer to the detection limit, despite the *LMP1* protein often being reported as an abundant component of EVs in other models [22], suggesting that post-transcriptional sorting mechanisms may decouple transcript and protein levels in this compartment.

Several limitations should be acknowledged. First, although EBV-negative multiple-myeloma cells do not represent an isogenic infection model, they provide a workable experimental framework to explore lipidomic differences associated with EBV latency in proliferating B-cell systems. Comparing multiple-myeloma-derived lines with LCLs introduces potential confounding factors inherent to their distinct oncogenic and differentiation backgrounds. Multiple myeloma represents a malignant plasma cell phenotype, whereas LCLs are shepherded by EBV toward a latently infected, proliferating mature B-cell state. Consequently, part of the observed lipidomic differences could reflect these divergent cellular identities rather than being exclusively driven by viral latency. However, the consistent enrichment of complex GSLs and the specific down-regulation of ceramides across all three independent EBV+ lines strongly echo established literature on EBV-mediated metabolic reprogramming, suggesting that the virus exerts a dominant, uniform pressure on sphingolipid pathways regardless of the baseline B-cell background.

Second, the extracellular material isolated by ultracentrifugation, while common and cost-effective [53,54], likely contains heterogeneous extracellular particles [55]. Third, additional EV-specific markers would be required to fully define vesicle subtypes according to MISEV guidelines [18]. Nevertheless, cryo-electron microscopy analysis was included to provide direct morphological visualization of extracellular particles enrichment of the isolated fraction. The small number of biological replicates per group and the absence of multiple comparison correction is a limitation; replication in larger, independent datasets will be required to confirm the reported associations.

## 5. Conclusions

In conclusion, our study has identified key sphingolipid species that define latently EBV-infected B-lymphocytes and provide an insight into morphology, viral cargo and sphingolipid profile of the released EPs.

We have shown a sphingolipid profile associated with EBV latency, characterized by GSL upregulation and an increased presence of pro-tumorigenic components associated with larger EPs.

Our results are in agreement with the current knowledge that EBV exploits host metabolism to sustain latency and transformation [7,14] and suggest that secretome-associated sphingolipids and viral transcripts represent an additional layer of metabolic and communicative adaptation used by the virus.

## Figures and Tables

**Table 1:** The ratios as log2 fold change in selected sphingolipids between EBV+ and EBV−cell lines, EBV+ and EBV− EPs; in the EBV+ group, the comparison between cell lines and EPs is reported. DHSph, dihydrosphingosine.

**EBV+Cells vs. EBV−Cells**	**∆ Fold Change**	***p*** **-Values**
Cer/S1P	1.2	0.0096
Cer/SM	−0.9	0.0721
Cer/DHSph	−3.8	0.0583
Cer/Sph	0.2	0.9069
Cer/Hexcer	−3.3	0.0427
Gb3/HexCer	3.6	0.0005
**EBV+EPs vs. EBV−EPs**	**∆ fold change**	***p*** **-values**
Cer/S1P	−0.8	0.9121
Cer/SM	1.6	0.0921
Cer/DHSph	1.2	0.9703
Cer/Sph	−1.0	0.0371
Cer/Hexcer	−2.4	0.0112
Gb3/HexCer	2.2	0.0002
**EBV+EPs vs. EBV+ cells**	**∆ fold change**	***p*** **-values**
Cer/S1P	−3.6	0.001
Cer/SM	−0.0	0.9269
Cer/DHSph	−1.3	0.9697
Cer/Sph	0.5	0.3388
Cer/Hexcer	−1.6	0.0216
Gb3/HexCer	0.4	0.0453

**Table 2:** Association between considered ratios and their biological role.

Ratio	Biological Role
Cer/SM	SM synthesis
Cer/HexCer	GSL synthesis
Gb3/HexCer	GSL synthesis
Cer/S1P	Cer degradation
Cer/Sph	Cer degradation
Cer/DHSph	Cer neo-synthesis

## Data Availability

The raw data for the LC-MS/MS are available in figshare, at the following link https://doi.org/10.6084/m9.figshare.32780460.

## Supplementary Materials

The following supporting information can be downloaded at: https://www.mdpi.com/article/10.3390/biology15161421/s1, Figure S1: structural and biophysical characterization of extracellular particles isolated by ultracentrifugation; Figure S2: western blot characterization of Extracellular particles; Supplementary Methods S1; Supplementary Results S1.

## Abbreviations

The following abbreviations are used in this manuscript:

Cer	Ceramide
DHCer	Dihydroceramide
DHSph	Dihydrosphingosine
EBV	Epstein–Barr virus
EPs	Extracellular particles
EVs	Extracellular vesicles
Gb3	Globotrialosylceramide
GSL	Glycosphingolipid
HexCer	Hexosylceramide
LacCer	Lactosylceramide
LC-MS/MS	Liquid chromatography tandem mass spectrometry
LCL	Lymphoblastoid cell line
nm	Nanometer
NPC	Nasopharyngeal carcinoma
NTA	Nanoparticle-tracking analysis
Sph	Sphingosine
S1P	Sphingosine-1-phosphate
SM	Sphingomyelin
sncRNA	Short non-coding RNA
