# Supplementary material for: Latent Epstein–Barr Virus Infection Correlates with Glycosphingolipid Enrichment and Morphological Remodeling of Extracellular Particles
Source: Biology (Basel). 2026 Aug 18;15(16):1421. doi: 10.3390/biology15161421 (PMC13509978; doi:10.3390/biology15161421)
Supplement: Supplementary file 1 [file biology-15-01421-s001.zip › Supplementary Methods S1.pdf]

SUPPLEMENTARY METHODS S1

Real Time Quantitative PCR (ZTA)

Cells were frozen in 700 uL of Qiazol (Qiagen) and stored at -80°C until extraction. The EBV positive control has been obtained from an infectious mononucleosis positive sample. mRNA extraction was performed with Rneasy MinElute cleanup kit (Qiagen) according to the manufacturer's instructions.

RNA was quantified with Nanodrop (Thermofisher) and cDNA was synthesized using GoScript (Promega, Madison, WI, USA) as per manufacturer's instructions.

RT-qPCR was performed using GoTaq® qPCR Master Mix (Promega) with 30ng of cDNA template per reaction. ZTA primers were obtained from SIGMA-ALDRICH used at a final concentration of 200nM. The 18S primers were designed as follow: 18S RNA-F, GTAACCCGTTGAACCCCATT; and 18S RNA-R, CCATCCAATCGGTAGTAGCG. The sequences are shown in the 5′–3′ direction. B-actin was purchased from BioRad (PrimePCR, Cat. n. 10025636). Amplification was carried out using FAST cycling protocol: 95°C for 2 minutes, 40 cycles of 95°C for 1 second, and 60°C for 30 seconds.

Cryo-electron microscopy (Cryo-EM)

Following the ultracentrifugation procedures, a sample containing 1×10^{10} extracellular vesicles (EVs) in 30 ml of DPBS was analyzed via cryo-electron microscopy at the Unitech NOLIMITS facility (University of Milano). Cryo-preservation was performed using a Vitrobot Mark IV (Thermo Fisher Scientific, USA). A volume of 3 µl was applied onto Quantifoil R 1.2/1.3 Cu 300-mesh grids that had been glow-discharged at 30 mA for 30 s with a GloQube system (Quorum Technologies). Following a 20-second incubation, the grids were blotted under controlled conditions (4°C, 100% relative humidity) and subsequently rapidly immersed in liquid ethane for vitrification. The frozen specimens were then loaded into a Talos Arctica transmission electron microscope (Thermo Fisher Scientific, USA), configured at 200 kV and with a Ceta 16M camera (Thermo Fisher Scientific, USA). Data were collected at a nominal magnification of 28,000x (pixel size: 0.370 nm/pixel).

Western blotting

EV protein extracts were prepared using RIPA buffer supplemented with protease and phosphatase inhibitor cocktails (Sigma-Aldrich, Italy). Protein content was quantified by Bradford assay. Equivalent protein amounts (20 µg) were resolved by SDS-PAGE on 15% gradient gels and then transferred onto nitrocellulose membranes (Hybond-ECL, Amersham Biosciences, Italy).

Membranes were incubated in blocking solution consisting of 5% non-fat milk in TBS-T buffer (20 mM Tris-Cl, pH 7.5, 150 mM NaCl, 0.05% Tween-20), followed by overnight incubation at 4°C with primary antibodies directed against TSG101 (Abcam, Ab83), CD81 (Cell Signaling Technology, #56039), CD63 (Cell Signaling Technology, #52090), and CD9 (Cell Signaling Technology, #99950) at a dilution of 1:1,000.

Following washing steps, membranes were incubated with species-specific HRP-conjugated secondary antibodies (Promega, USA). Signal detection was performed using an enhanced chemiluminescence substrate (WesternBright ECL, Advansta Inc., USA), and images were acquired with a ChemiDoc Imaging System (Bio-Rad Laboratories, USA). Western blot analyses were independently performed twice.
