# Supplementary material for: Latent Epstein–Barr Virus Infection Correlates with Glycosphingolipid Enrichment and Morphological Remodeling of Extracellular Particles
Source: Biology (Basel). 2026 Aug 18;15(16):1421. doi: 10.3390/biology15161421 (PMC13509978; doi:10.3390/biology15161421)
Supplement: Supplementary file 1 [file biology-15-01421-s001.zip › Supplementary Results S1.pdf]

SUPPLEMENTARY RESULTS S1

ZTA qPCR expression evaluation

The results of the qPCR for the identification of ZTA are showed as Mean Ct values from the qPCR.

Sample	Target	Ct Mean
**ARH77**	Beta-Actin	28.26708603
	18S	27.82967377
	ZTA	Undetermined
**CN1208550M**	Beta-Actin	17.14974976
	18S	18.39707375
	ZTA	38.7110939
**CN50271**	Beta-Actin	17.45169258
	18S	19.21586037
	ZTA	39.03409958
**EBV+**	Beta-Actin	15.0881958
	18S	29.69930077
	ZTA	22.5226593
